# Shedding of a Low Pathogenic Avian Influenza Virus in a Common Synanthropic Mammal – The Cottontail Rabbit

**DOI:** 10.1371/journal.pone.0102513

**Published:** 2014-08-11

**Authors:** J. Jeffrey Root, Susan A. Shriner, Kevin T. Bentler, Thomas Gidlewski, Nicole L. Mooers, Terry R. Spraker, Kaci K. VanDalen, Heather J. Sullivan, Alan B. Franklin

**Affiliations:** 1 United States Department of Agriculture, Wildlife Services, National Wildlife Research Center, Fort Collins, Colorado, United States of America; 2 Department of Microbiology, Immunology, and Pathology, Colorado State University, Fort Collins, Colorado, United States of America; German Primate Center, Germany

## Abstract

**Background:**

Cottontails (*Sylvilagus* spp.) are common mammals throughout much of the U.S. and are often found in peridomestic settings, potentially interacting with livestock and poultry operations. If these animals are susceptible to avian influenza virus (AIV) infections and shed the virus in sufficient quantities they may pose a risk for movement of avian influenza viruses between wildlife and domestic animals in certain situations.

**Methodology/Principal Findings:**

To assess the viral shedding potential of AIV in cottontails, we nasally inoculated fourteen cottontails with a low pathogenic AIV (H4N6). All inoculated cottontails shed relatively large quantities of viral RNA both nasally (≤10^6.94^ PCR EID_50_ equivalents/mL) and orally (≤10^5.09^ PCR EID_50_ equivalents/mL). However, oral shedding tended to decline more quickly than did nasal shedding. No animals showed any obvious signs of disease throughout the study. Evidence of a serological response was found in all infected rabbits at 22 days post infection in convalescent sera.

**Conclusions/Significance:**

To our knowledge, cottontails have not been previously assessed for AIV shedding. However, it was obvious that they shed AIV RNA extensively via the nasal and oral routes. This is significant, as cottontails are widely distributed throughout the U.S. and elsewhere. These mammals are often found in highly peridomestic situations, such as farms, parks, and suburban neighborhoods, often becoming habituated to human activities. Thus, if infected these mammals could easily transport AIVs short distances.

## Introduction

Influenza A viruses are globally important public health and veterinary pathogens infecting numerous avian and mammalian species [1]. These viruses have produced large financial burdens in terms of public health [2] and poultry production [3]. Wild birds of the orders Anseriformes and Charadriiformes are typically considered the primary natural hosts of avian influenza viruses (AIVs) [4]. Despite documented associations of influenza A viruses and wild mammals, the potential role of these species in the ecology of influenza A viruses has received limited attention and only for select species [5]–[10].

While infections of mammals with highly pathogenic (HP) Asian strain H5N1 AIV have been most commonly found in species from the mammalian order Carnivora, a few exceptions have been noted. One recent exception occurred in the mammalian order Lagomorpha (e.g., hares, rabbits, and pikas) where 13.4% of 82 wild black-lipped pika (*Ochotona curzoniae*) had antibodies against HP H5N1 in and around Qinghai Lake, China [10]. In addition, 5 viral isolates of HP H5N1 were obtained from tissues of this species from the same region [10]. While no naturally occurring infections of HP AIV H5N1 have been reported in rabbits [11], the presence of this virus in black-lipped pika represents the first cases of HP H5N1 documented in a lagomorph [10], [11]. To our knowledge, no other natural AIV infections have been reported in lagomorphs.

Cottontails (*Sylvilagus* spp.) occur as multiple species in North America and are broadly distributed throughout the United States [12]. The desert cottontail (*Sylvilagus audubonii*) has an expansive distribution in western North America, ranging from northern Montana, south to central Mexico, and west to southern California [13]. It is well-adapted for a diversity of habitats [14], is not territorial, and forages primarily on forbs and grasses [15]. Importantly, cottontails are frequently found in peridomestic situations, often living within farmsteads, commercial properties, parks, and suburban neighborhoods. They are also commonly found in other areas associated with metropolitan landscapes in parts of the U.S. [16]. Thus, cottontails, to a large extent, are synanthropic. These habits, in the context of biosecurity, may be even more important if one considers an avian-rearing facility, as wild mammals have been documented near bird production areas [17].

Interactions among domestic poultry and other animals have been suggested as a potential pathway of avian pathogen introductions for domestic poultry flocks [18]. For example, multiple conduits of exposure of AIVs through wild birds have been verified or alleged in causing outbreaks in poultry [1]. Other species, such as wild mammals, have also been implicated as risk factors associated with the spread of a low pathogenicity (LP) AIV among commercial poultry farms [19]. Given that at least one lagomorph species was naturally susceptible to HP H5N1 [10], this mammalian order warrants more scrutiny for its potential role in AIV ecology. Cottontails, which range throughout much of the U.S., are an obvious choice to further assess the competency of synanthropic lagomorphs to shed AIV. The objective of this study was to assess the shedding potential of cottontails experimentally infected with a LP AIV (H4N6), an AIV frequently found in wild waterfowl in North America [20]. In meeting this objective, we addressed three research questions: what is the magnitude and duration of AIV shedding in cottontails, what are the primary routes of AIV shedding, and how consistent were these characteristics across individuals?

## Methods

### Ethics Statement

Animal experiments were approved by the Institutional Animal Care and Use Committee of the National Wildlife Research Center (NWRC), Fort Collins, CO, USA (Approval number 1807). Cottontails were captured on state-owned land with facility manager permission under a state collection permit issued by the Colorado Division of Wildlife.

### Study animals

Cottontails were live-trapped in box-style traps (15.2×15.2×48.3 cm; Tomahawk Live Traps, LLC, Hazelhurst, WI, USA) in Larimer County, Colorado. Upon capture, pre-experiment blood and nasal samples were obtained. In addition, all animals were dusted for ecto-parasites and individually marked with microchips. Cottontail species were identified as desert cottontails (*Sylvilagus audubonii*) using methods described elsewhere [21]. A total of sixteen desert cottontails were used in the experiment outlined below. The sixteen cottontails were randomly assigned to one of two groups: odd or even.

For quarantine purposes, cottontails were housed in an outdoor animal research building in customized 58.4×66.0×91.4 cm dog crates (Precision Pet Products, Costa Mesa, CA, USA). Each crate was outfitted with a hide made from PVC tube with a 20 cm inner diameter, a water bowl, a food dish, a hay bowl, and an enrichment toy. Food (Purina Rabbit Chow [Purina Mills, St. Louis, MO, USA], alfalfa, and apples or carrots) and water were replenished daily. Following a minimum of a 14-day quarantine period, all rabbits were transferred to a BSL-2 animal facility and housed individually in 59.7×40.6×45.7″ rabbit racks outfitted with the same materials as the dog crates. The control animals were maintained in a separate rack within the same animal room.

### Experimental Infection

On day 0 of this experiment, fourteen animals were anesthetized with isoflurane vaporizers and nasally inoculated with approximately 10^5.4^ EID_50_ of a LP AIV H4N6 diluted in 250 µL of BA-1 viral transport medium (see [8] for formula). Details of the virus have been presented elsewhere [22]. The control cottontails (n = 2) received 250 µL mock inoculations of BA-1 containing no virus.

For sampling from 1–8 days post infection (DPI), eight cottontails were sampled on odd days and eight cottontails were sampled on even days so that each animal was processed every other day to limit handling stress. All animals were also sampled on 16 and 22 DPI. Prior to sampling, all animals were anesthetized with isoflurane. Daily processing consisted of a nasal wash, an oral swab, and the collection of a fecal pellet from each individual sampled. Swabs and fecal pellets were stored in 1 mL of BA-1 medium and nasal cavities were washed using 1 mL of BA-1. All samples were stored on ice packs in animal rooms and were transferred to −80°C freezers immediately following the conclusion of daily processing. On 22 DPI, blood was collected and all animals were humanely euthanized.

### Necropsy and Tissue Processing

Following euthanasia, animals were examined. Gross lesions were not observed in any of the cottontails. Tissues from major organs were collected for histopathology and preserved in 10% neutral buffered formalin. Fixed tissues were trimmed, placed in cassettes, processed overnight (Sakura Tissue-Tek VIP 6), embedded in paraffin, sectioned at 5 um and stained with hematoxylin and eosin. In addition, approximately 75 mg of nasal turbinates, trachea, upper lung lobe, lower lung lobe, and colon were collected into vials with 1 mL BA1 and homogenized and centrifuged as previously described [8] for testing by real-time reverse-transcription polymerase chain reaction (RRT-PCR). Animal carcasses were incinerated following necropsy procedures.

### Laboratory Testing

Fecal pellets, oral swabs, and nasal washes were tested in duplicate by RRT-PCR for viral RNA detection and quantification. Primers and protocols [23], [24], along with protocol modifications have been described in detail elsewhere [25]. Consistent with a previous study, positive samples were defined as those yielding a two-well positive amplification with a Ct value of ≤38 and suspect positive samples were defined as those yielding a two-well positive amplification with a Ct value of >38 [25]. Negative samples were defined as those yielding no Ct value or one that amplified a single well. Calibrated control samples were also analyzed with RRT-PCR to construct standard curves for each run. Viral RNA quantities from samples were extrapolated from the standard curves and are presented as PCR EID_50_ equivalents/mL. Details about this procedure have been published elsewhere [25]. In addition, select nasal wash (2 and 7 DPI) and oral swab samples (2 and 5 DPI) were tested for live virus by virus isolation in embryonated chicken eggs following published protocols [26].

Serum samples collected pre- and post-inoculation were tested for anti-influenza virus A antibodies via the FlockCheck Avian Influenza MultiS-Screen Antibody Test Kit (IDEXX Laboratories, Inc., Westbrook, ME) and Agar Gel Immunodiffusion (AGID) [27], [28].

## Results

### Nasal Shedding

**Figure 1:**
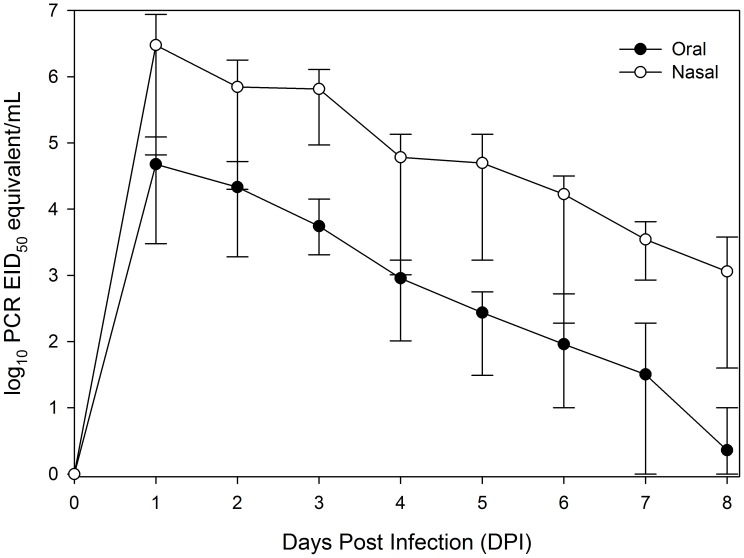
Mean nasal and oral shedding of avian influenza virus RNA of desert cottontails experimentally infected with a low-pathogenic avian influenza virus. Shedding was assessed from nasal washes and oral swabs by RRT-PCR. Results are presented as log_10_ PCR EID_50_ equivalents/mL. Vertical bars represent the maximum and minimum quantities detected on a given day.

All inoculated animals yielded a minimum of 10^4.8^ PCR EID_50_ equivalent/mL from nasal washes during the first DPI they were sampled (1 or 2 DPI; Figure 1). Nasal shedding peaked on 1 DPI yielding an average of 10^6.47^ PCR EID_50_ equivalent/mL (range = 10^4.82^ to 10^6.94^; Figure 1). With one exception, all treatment cottontails yielded their highest nasal wash on the first day they were sampled. As expected, a declining trend in viral RNA was noted during 1–8 DPI. By 16 DPI, eight of fourteen (57.1%) test animals were suspect positive. At 22 DPI, a single cottontail still had evidence of nasal shedding of viral RNA. The nasal washes from the two control cottontails remained negative throughout the study. All nasal washes tested positive for live virus during 2 DPI; however, this figure was greatly reduced by 7 DPI, as a single nasal wash tested positive for live virus at this time point.

### Oral Shedding

All treatment cottontails showed evidence of oral shedding of AIV RNA by one or two DPI (Figure 1). Similarly to nasal washes, oral shedding peaked on the first day sampled with an average of 10^4.68^ PCR EID_50_ equivalent/mL (range = 10^3.48^ to 10^5.09^; Figure 1). By 8 DPI, all but one treatment animals were negative. By 16 DPI, only one individual was suspect positive, which was a different individual than that sampled during 8 DPI. At 22 DPI, all oral swabs were negative. Both control animals yielded negative results throughout the study. All oral swabs tested positive for live virus during 2 DPI, while five of seven tested positive for live virus on 5 DPI.

### Fecal Shedding

Viral RNA was rarely detected in or on the fecal pellets of cottontails during 2 to 4 DPI. Positive results (n = 2) of approximately 10^2.0^ PCR EID_50_ equivalent/mL were noted on 2 DPI, while suspect positive results were noted on 2, 3, and 4 DPI. The control animals remained negative throughout the experiment. It should be noted, however, that the positive results we observed may simply be a result of contamination by oral or nasal secretions.

### Individual Variation

Although all inoculated cottontails shed during this study, as expected, individual heterogeneity (variation among individuals) was commonly observed from both the nasal and oral routes of shedding (Figure 1). However, no clear pattern associated with DPI was noted.

### RNA Detection in Tissues

Select tissues (nasal turbinates, trachea, lung [lower and upper lobes], and colon) were collected during necropsy on 22 DPI for RRT-PCR analyses. Two trachea samples yielded the only positive results. However, all other samples types yielded at least one suspect positive result, which suggests that in general this virus primarily cleared in this species within or earlier than three weeks of infection.

### Serology

Although the FlockCheck Avian Influenza MultiS-Screen Antibody Test Kit has been evaluated for some mammal species [29], this test did not appear to work with our cottontail sera. However, all inoculated cottontails yielded evidence of a serological response in their convalescent sera at 22 DPI based on AGID. Serum samples from the 14 treatment cottontails were scored as strong positive (78.6%; *n* = 11) or positive (21.4%; *n* = 3). The 22 DPI serum samples from the control animals were scored as negative.

### Pathology

Histological lesions were not found in the two control cottontails. A mild, subacute lymphoblastic tracheitis was found in two animals and a mild, multifocal subacute lymphoplasmocytic pneumonia characterized by mild accumulations of lymphocytes and plasma cells around vessels and bronchioles was found in four cottontails. One of these cottontails also had a mild bronchitis.

## Discussion

We detected high titers of AIV RNA in nasal secretions as early as 1 DPI in cottontails experimentally infected with LP H4N6 AIV, but all animals shed significantly less quantities by 8 DPI (Figure 1). In contrast, New Zealand white rabbits experimentally infected with HP H5N1 AIV of pika origin (A/PK/QH/BI/0704/2007 and A/PK/QH/QW/0712/2007) initiated nasal shedding of virus at 3 DPI and all shedding ceased by 10 DPI or earlier [10]. However, the same rabbit species experimentally infected with HP H5N1 (A/chicken/Hong Kong/220/97) did not yield any evidence of a productive infection [30]. The high levels of nasal shedding observed on 1 DPI during the current study could potentially be due, in part, to some residual inoculum. As such, these early titers should be interpreted with caution.

Strong evidence of oral shedding of viral RNA was noted in cottontails during the present study (Figure 1). Titers remained relatively high through 4 DPI, but decreased during subsequent DPI. In contrast, virus was not detected in oropharyngeal swabs from New Zealand white rabbits experimentally infected with HP H5N1 [10]. Combined, these data suggest very different shedding patterns among these different, but related lagomorph species infected with various subtypes of HP and LP AIVs.

The high levels of nasal shedding of AIV RNA by cottontails in this study by 1 DPI was somewhat surprising and raises the question of the potential of residual virus from nasal inoculations affecting the titers we observed. However, other studies have detected AIVs in different mammal species as early as 1 DPI [31].

It has been suggested that proximity to and contact with avian reservoirs are likely important elements that may have facilitated cross-species transmission of LP AIV to various mammalian species [32]. These factors are also likely linked to HP AIV infections in mammals. For example, it has been hypothesized that black-lipped pika, a close relative of rabbits, were exposed to HP H5N1 through shared vegetative foraging sites with birds [10]. A similar scenario might be plausible for cottontails, as cottontails could utilize the same foraging sites as wild birds and acquire an AIV infection via environmental contamination. Similarly, this scenario is possible in poultry rearing facilities with insufficient biosecurity, as a cottontail could acquire an AIV infection in contaminated bird pens or potentially transmit AIV through shared feed. Notably, bird to mammal transmission of AIV, likely through environmental contamination, has recently been documented in an experimental setting [9].

Host species barriers can limit the cross-species transmission of AIV to mammals [32], [33]. As such, a myriad of factors such as virus-host interactions (e.g., within host barriers) and host-host interactions (e.g., cross-species contacts) are thought to be important requisites for cross-species transmission [33]. We have established that cottontails are effectively infected with AIV via the nasal route and subsequently shed viral RNA for several days. In addition, the histological lesions found in five of fourteen infected cottontails suggested that the virus we studied did invade pulmonary tissues but caused minimal tissue damage that was likely reversible. However, effective natural exposures of these animals to AIVs along with their ability to transmit the virus to a new host are undetermined at this time. Thus, multiple barriers may limit the effective host range of AIV in the mammal species we studied [32]. However, in the case of wild peridomestic mammals, a single infection that does not spread to conspecifics could pose some risk, as the mammal could transfer the virus to more susceptible avian species in operational settings.

Overall, this study suggests that at least one species of cottontail is susceptible to AIV infection and sheds relatively large quantities of viral RNA through both the nasal and oral routes. The shedding potential of cottontails, coupled with their synanthropic habits and their ability, in some instances, to move from pen-to-pen in production facilities with limited biosecurity suggests that this species could pose a threat to poultry operations. Additional studies are needed to assess the natural exposure routes of AIVs in cottontails to assess if and how frequently cottontails are exposed to these viruses in natural settings.
